# Effect of Phosphatase and Tensin Homologue on Chromosome 10 on Angiotensin II-Mediated Proliferation, Collagen Synthesis, and Akt/P27 Signaling in Neonatal Rat Cardiac Fibroblasts

**DOI:** 10.1155/2016/2860516

**Published:** 2016-09-25

**Authors:** Ling Nie, Jing-Hong Zhao, Jiang Wang, Rong Song, Shan-Jun Zhu

**Affiliations:** ^1^Department of Nephrology, Xinqiao Hospital, The Third Military Medical University, Chongqing 400037, China; ^2^Department of Cardiovascular Disease, Xinqiao Hospital, The Third Military Medical University, Chongqing 400037, China

## Abstract

Cardiac fibroblasts (CFs) play a key role in cardiac fibrosis by regulating the balance between extracellular matrix synthesis and breakdown. Although phosphatase and tensin homologue on chromosome 10 (PTEN) has been found to play an important role in cardiovascular disease, it is not clear whether PTEN is involved in functional regulation of CFs. In the present study, PTEN was overexpressed in neonatal rat CFs via recombinant adenovirus-mediated gene transfer. The effects of PTEN overexpression on cell-cycle progression and angiotensin II- (Ang II-) mediated regulation of collagen metabolism, synthesis of matrix metalloproteinases, and Akt/P27 signaling were investigated. Compared with uninfected cells and cells infected with green fluorescent protein-expressing adenovirus (Ad-GFP), cells infected with PTEN-expressing adenovirus (Ad-PTEN) significantly increased PTEN protein and mRNA levels in CFs (*P* < 0.05). The proportion of CFs in the G1/S cell-cycle phase was significantly higher for PTEN-overexpressing cells. In addition, Ad-PTEN decreased mRNA expression and the protein synthesis rate of collagen types I and III and antagonized Ang II-induced collagen synthesis. Overexpression of PTEN also decreased Ang II-induced matrix metalloproteinase-2 (MMP-2) and tissue inhibitor of metalloproteinase-1 (TIMP-1) production as well as gelatinase activity. Moreover, Ad-PTEN decreased Akt expression and increased P27 expression independent of Ang II stimulation. These results suggest that PTEN could regulate its functional effects in neonatal rat CFs partially via the Akt/P27 signaling pathway.

## 1. Introduction

Cardiac remodeling is a complex process that involves ultrastructural rearrangement of the heart [1, 2]. Cardiac fibrosis plays an important role in this process by adversely affecting systolic and diastolic functions [3, 4]. Emerging evidence suggests that cardiac fibroblasts (CFs) regulate the balance between synthesis and breakdown of extracellular matrix proteins during cardiac fibrosis [3, 5]. Although CFs synthesize several interstitial proteins (e.g., collagens) in the heart and release enzymes such as matrix metalloproteinases (MMPs) and tissue inhibitors of metalloproteinases (TIMPs), the detailed mechanisms responsible for regulating the function of CFs are not fully understood [6].

Phosphatase and tensin homolog on chromosome ten (PTEN) is a 3′-lipid phosphatase that is widely expressed in various cell types including cardiomyocytes, vascular smooth muscle cells (VSMCs), and endothelial cells [7–9]. Parajuli et al. found that PTEN regulates cardiac remodeling after myocardial infarction via the Akt/interleukin-10 signaling pathway [10]. Moreover, cardiac-specific PTEN inactivation protects the heart from functional failure in a mouse model of pressure overload [10]. PTEN-inducible kinase 1 (PINK1) is downregulated in patients with end-stage heart failure, and PINK1(−/−) mice exhibit elevated oxidative stress, impaired mitochondrial function, increased fibrosis, ventricular dysfunction, and cardiac hypertrophy [11]. PTEN expression is upregulated in VSMCs following in vivo and in vitro vascular injury. PTEN upregulation worsens H_2_O_2_-induced apoptosis by altering Akt-dependent signaling [12]. Although these findings indicate a crucial role of PTEN in cardiac function, the cellular effects of PTEN in functional regulation of CFs remain unclear.

Angiotensin II (Ang II) regulates collagen synthesis and production and promotes cardiac fibrosis [13, 14]. Whether PTEN mediates its effects in CF in an Ang II dependent manner is also unknown.

In the present study, recombinant adenovirus-mediated gene transfer was used to enhance PTEN expression over basal levels in neonatal rat CFs so as to study the effects of PTEN on Ang II-induced CF proliferation, apoptosis, cell cycle, and collagen metabolism.

## 2. Materials and Methods

### 2.1. Cell Culture

Animal experiments were conducted in accordance with guidelines established by the Animal Care and Use Committee of The Third Military Medical University.

CFs were isolated from the left ventricles of 3-day-old Sprague-Dawley rats, as previously described [15]. In brief, hearts were harvested from 1–3-day-old SD rats after they were anesthetized with ether and disinfected with alcohol. The hearts were then minced in D-Hank's buffer (116 mM NaCl, 20 mM HEPES, 9.4 mM NaH_2_PO_4_, 5.5 mM glucose, 5.4 mM KCl, and 0.4 mM MgSO_4_, pH 7.4). The left ventricular tissue was digested at 37°C with 0.16% trypsin and 0.06% collagenase in sterile D-Hank's buffer for 9 min. The digestion was repeated five times. Next, the cells were centrifuged at 1000 ×g for 10 min, resuspended in Dulbecco's modified Eagle medium (DMEM) containing 10% fetal bovine serum, 100 U/mL penicillin, and 100 U/mL streptomycin, and seeded on a 10 cm culture dish at a concentration of 10^6^ cells/mL. After preincubation at 37°C for 90 min, CFs were allowed to differentiate from cells that adhered to the culture dish. The medium was replaced with DMEM supplemented with 10% fetal bovine serum, and cells were cultured in a 5% CO_2_ incubator at 37°C for 48 h. The purity of the cultured fibroblasts was determined on the basis of positive staining for vimentin and fibronectin and negative staining for *α*-sarcomeric actin. Cells from the second through fourth passages were included for all experiments.

### 2.2. Plasmid Construction and Recombinant Adenovirus Generation

Adenovirus vector carrying the wild-type PTEN gene (from human species) was constructed by cloning and homologous recombination. An adenovirus vector carrying the green fluorescent protein (GFP) reporter gene was used as the negative control. Recombinant adenoviruses were produced by transfection of homologous recombinant plasmid into the AD-293 cell line.

### 2.3. Infection of Cultured Fibroblasts with Recombinant Adenoviruses

Fibroblasts were cultured in a 250 mL culture bottle containing complete medium and were infected with adenoviruses at 90% confluency. The multiplicity of infection (MOI) was calculated using the following equation: MOI (pfu/mL) = [number of GFP positive cells × virus dilution (GFP positive maximum dilution)]/0.1 mL. To ensure comparable multiplicity of infection (MOI) between passages with different growth rates, cells plated on 96-well culture dishes at a concentration of 10^5^ cells/mL were trypsinized and counted. Adenovirus (MOI = 100) was resuspended in 0.5 mL of DMEM and added to each well immediately after aspirating the medium. Complete medium (1.5 mL) was added to the wells after 2 h. After 48 h of incubation, fibroblasts were treated with Ang II (10^−7^ mM) or DMEM for 24 h. Fibroblasts were divided into the following treatment groups: Control (DMEM), Ad-GFP, PTEN adenovirus (Ad-PTEN), Control+Ang II, Ad-GFP + Ang II, and Ad-PTEN + Ang II. Each experiment was repeated three times with 3-4 replicates each.

### 2.4. Reverse Transcription-Polymerase Chain Reaction (RT-PCR)

Total RNA was isolated from CFs using the Tripure reagent (Roche, Basel, Switzerland), according to the manufacturer's instructions. RNA concentration and purity were measured using a UV spectrophotometer. Subsequently, first-strand cDNA was obtained from total RNA (1000 ng) using the M-MLV reverse transcriptase (Promega, Madison, WI, USA) and random hexamer primers. PCR amplification of specific genes was conducted using a prealiquoted PCR master mix (TOYOBO, Japan) in a thermocycler (T100*™*, Bio-Rad, USA). The PCR cycle conditions were 94°C for 2 min, followed by 40 cycles of 94°C for 10 s, 55°C for 30 s, and 68°C for 60 s each, and finally one cycle of 68°C for 5 min. PCR products were separated by agarose gel (1.5%) electrophoresis and stained with ethidium bromide. Gels were scanned using a Nucleo Vision imaging workstation (NucleoTech, San Mateo, CA, USA), and bands were quantified using the GelExpert release 3.5 software. The results are reported as mean band densities of various genes relative to the band intensities of GAPDH as the normalization control. The respective primers and PCR product specifications are listed in Table 1.

### 2.5. Western Blotting

CFs from each group at various time points (approximately 10^7^ cells) were washed in cold phosphate-buffered saline (PBS) and lysed in RIPA lysis buffer (Beyotime Institute of Biotechnology) containing 50 mM Tris-HCl (pH 7.4), 150 mM NaCl, 1% Triton X-100, 1% sodium deoxycholate, 0.1% sodium dodecyl sulfate (SDS), 5 mM NaF, 1 mM phenylmethylsulfonyl fluoride, 1 mM sodium orthovanadate, 1 mM EDTA, 1 mM EGTA, and complete protease inhibitor mixture for 20 min on ice, according to the manufacturer's instructions. Protein concentration was measured using the bicinchoninic acid protein assay kit (Bio-Rad, Hercules, CA, USA), with bovine serum albumin as the standard. Equal amounts of protein extracts (2 mg/mL) were mixed with SDS-polyacrylamide gel electrophoresis (PAGE) sample buffer and boiled at 95–100°C for 5 min. The samples were then separated on 10% SDS-PAGE gels and transferred onto polyvinylidene difluoride membranes (Millipore, Billerica, MA, USA). Nonspecific binding sites were blocked by incubating the membranes with 5% nonfat dry milk for 2 h at room temperature in Tris-buffered saline with Tween (TBS-T) [20 mM Tris-HCl (pH 8.0), 8 g/L NaCl, and 0.1% Tween 20]. The blots were washed in TBS-T three times for 10 min and incubated at 4°C overnight with the appropriate primary antibody: mouse anti-GAPDH (1 : 1000 dilution; KangChen Bio-tech Inc., Shanghai, China); mouse anti-PTEN (1 : 1000 dilution; Cell Signaling Technology, Boston, MA, USA); rabbit anti-P27 (1 : 1000 dilution; Cell Signaling Technology, Boston, MA, USA); or rabbit anti-Akt (Thr-308 phosphorylation site, 1 : 1000 dilution; Santa Cruz Biotechnology, Santa Cruz, CA, USA).

The blots were then washed with TBS-T and incubated with horseradish peroxidase-conjugated anti-mouse (1 : 1000 dilution; KangChen Bio-tech Inc., Shanghai, China) or anti-rabbit (1 : 1000 dilution; Cell Signaling Technology, Boston, MA, USA) secondary antibodies diluted in 5% nonfat dry milk for 2 h at room temperature. After washing the membranes thrice in TBS-T, the proteins were detected by the 3,3′-diaminobenzidine colorimetric method (Boster Biological Engineering Co., Ltd. Wuhan, China), according to the manufacturer's instructions. Each experiment was performed in triplicate and repeated three times. Immunoreactive bands were quantified by using the Quantity One densitometer analysis system (model 4.6.2, Bio-Rad, USA). The protein levels in each sample were normalized to the level of GAPDH protein.

### 2.6. Analysis of Cell Cycle Distribution

Fibroblasts were cultured in 250 mL culture bottles for 24 h, after which they were washed once in PBS and detached by adding 0.25% trypsin. The cells were resuspended in complete DMEM and centrifuged at 200 ×g for 5 min. Next, the cells were fixed in 80%–95% ethanol and stored at 4°C. The cell-cycle status was then measured by staining CFs with propidium iodide (50 mg/mL in PBS containing 0.1% Triton X-100) at 4°C. After staining for at least 24 h, flow cytometry was performed on a FACScan instrument (BD Biosciences), and data were analyzed using the CellQuest.

### 2.7. Total Collagen Assay

The ^3^H-proline incorporation assay was used to determine collagen synthesis, as previously described [16].

### 2.8. Gelatin Zymography

Gelatinase activity was detected by gelatin zymography, according to a previous study [17]. Gelatinase activity was calculated using the following equation: Gelatinase activity = area of band × (band intensity − background intensity)/concentration of sample protein.

### 2.9. Statistical Analysis

Data were presented as mean ± standard deviation. The SPSS 16.0 software for Windows (SPSS, Chicago, IL, USA) was used for statistical analysis. The normal distribution of data was checked by the SPSS program. For comparison of different groups, the unpaired Student's *t*-test and one-way analysis of variance with repeated measures were used, where appropriate. Significance was set at *P* < 0.05.

## 3. Results

### 3.1. Overexpression of PTEN in CFs

We used cultured fibroblasts with >95% purity for all experiments. We first established the optimal MOI and time for infection of adenovirus on the basis of GFP expression in CFs transfected with the Ad-GFP plasmid by fluorescence microscopy. At an MOI of 100, the positive rate of GFP expression exceeded 90%, with no further increase at MOI > 100. With regard to time course, maximum GFP expression was observed at 48 h. Hence, all subsequent analyses were performed at an MOI of 100 and a transfection time of 48 h.

**Figure 1:**
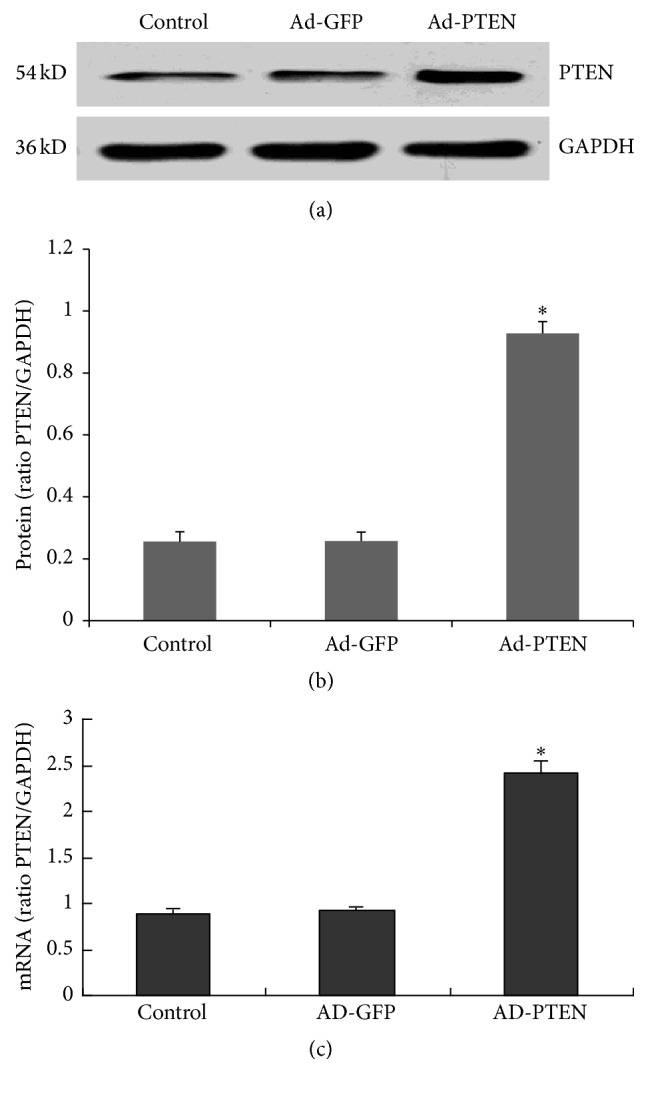
PTEN overexpression in cardiac fibroblasts (CFs) by adenoviral gene transfer. Infection with Ad-GFP or Ad-PTEN for 48 h. (a, b) PTEN protein expression was assessed by western blot analysis. (c) The PTEN mRNA level was assessed by semiquantitative RT-PCR. Each experiment was repeated three times with 3-4 replicates each. ^*∗*^ *P* < 0.05, compared with the control Ad-GFP and uninfected groups.

PTEN protein expression was assessed by western blotting (Figures 1(a) and 1(b)). Compared with PTEN expression in uninfected cells and cells infected with the Ad-GFP virus, PTEN expression was significantly higher in cells infected with Ad-PTEN virus at an MOI of 100 for 48 h. PTEN mRNA levels of cells treated with Ad-PTEN were also significantly increased as observed by semiquantitative RT-PCR analysis (Figure 1(c)).

### 3.2. Effect of PTEN Overexpression on the Cell Cycle in CFs

**Figure 2:**
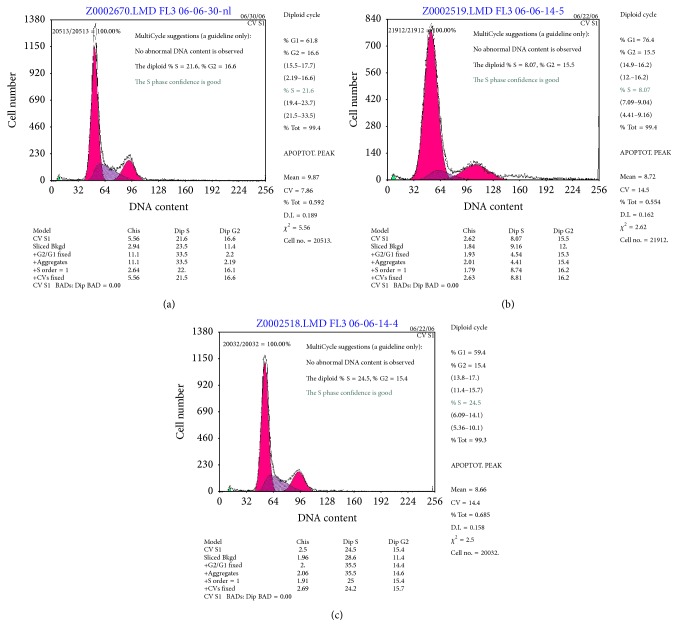
Effect of PTEN overexpression on the cell cycle in CFs. Infection with Ad-GFP or Ad-PTEN for 48 h. The cell cycle phases were analyzed by flow cytometry: (a) uninfected cells; (b) control Ad-GFP-treated cells; and (c) Ad-PTEN-infected cells. Each experiment was repeated three times with 3-4 replicates each.

Compared with the uninfected cells, cells infected with Ad-GFP virus for 48 h showed no changes in the distribution of cell-cycle phases (Figures 2(a) and 2(b)). However, the proportion of CFs in the G1/S phase was significantly increased in the Ad-PTEN-infected cells (Figure 2(c), Table 2), suggesting a block at the G1/S stage with PTEN overexpression.

### 3.3. PTEN Overexpression Inhibits Ang II-Induced Collagen Synthesis

**Figure 3:**
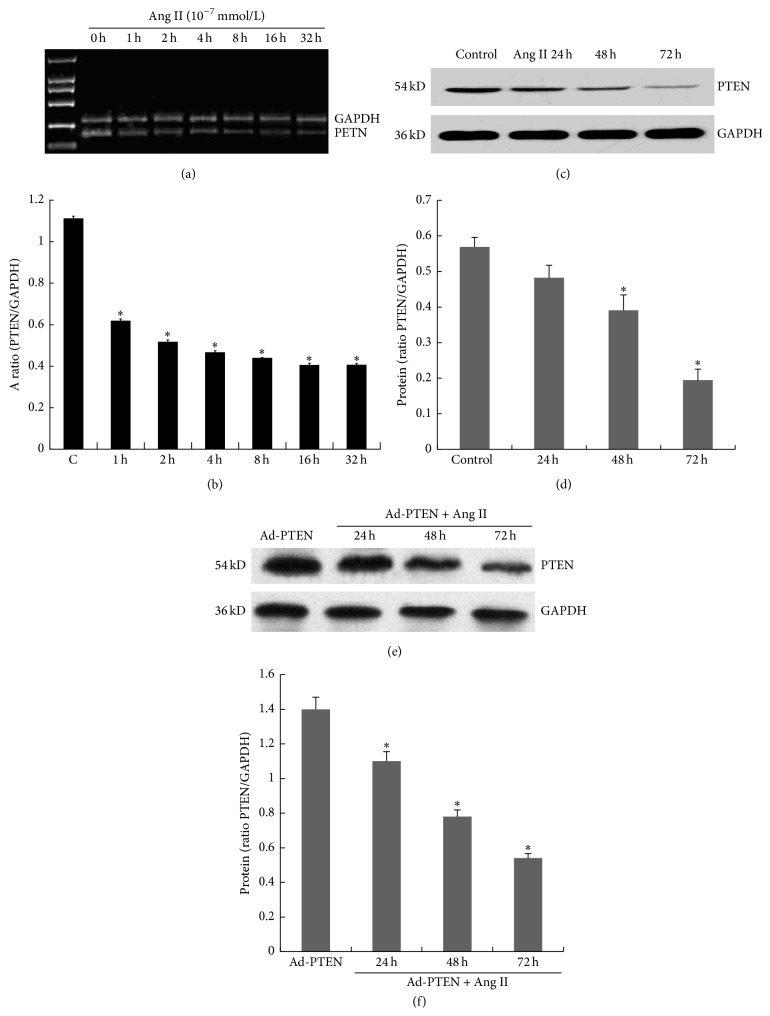
Effect of angiotensin II (Ang II) on PTEN expression in CFs. Fibroblasts were treated with Ang II (10^−7^ mM) for 32 h. (a, c) The PTEN mRNA level was assessed by RT-PCR. (b, d, e, f) PTEN protein expression was assessed by western blotting. Each experiment was repeated three times with 3-4 samples. ^*∗*^ *P* < 0.05 compared with the control group.

**Figure 4:**
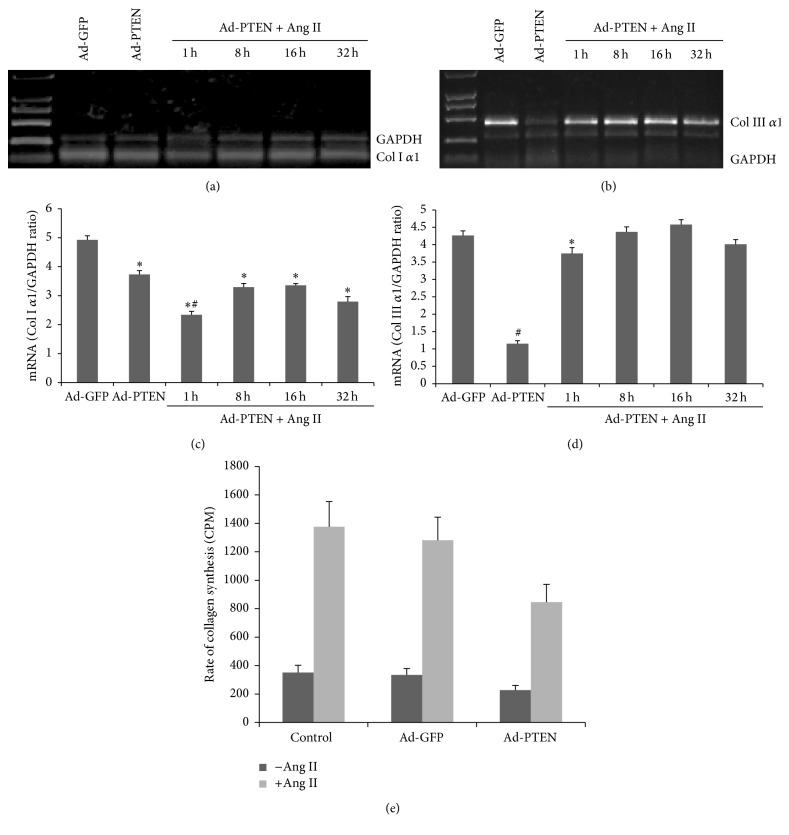
Overexpression of PTEN inhibits Ang II-induced collagen synthesis in CFs. After infection for 48 h, fibroblasts were treated with Ang II (10^−7^ mM) for 32 h. (a, b) The collagen type I-*α*1 mRNA level was assessed by semiquantitative RT-PCR. (c, d) The collagen type III-*α*1 mRNA level was assessed by semiquantitative RT-PCR. (e) The protein synthesis rate of collagen was assessed by ^3^H-proline incorporation assay. Each experiment was repeated three times with 3-4 replicates. ^*∗*^ *P* < 0.05, compared with the control Ad-GFP and untreated groups. ^#^ *P* < 0.05, compared with Ad-GFP + Ang II.

Compared with the uninfected cells, Ang II (10^−7^ mM) stimulation decreased PTEN mRNA levels at 1, 2, 4, 8, 16, and 32 h (Figures 3(a) and 3(c)) and protein expression at 48 and 72 h, but not at 24 h (Figures 3(b) and 3(d)). Compared with Ad-GFP infected cells, Ang II (10^−7^ mM) stimulation decreased PTEN expression gradually at 24, 48, and 72 h (Figures 3(e) and 3(f)). Compared with the uninfected and Ad-GFP infected cells, Ad-PTEN-infected cells had decreased collagen type I (ColI *α*1, Figures 4(a) and 4(c)) and collagen type III (Col III *α*1, Figures 4(b) and 4(d)) mRNA levels. Furthermore, that rate of collagen synthesis was significantly different in Ad-PTEN-infected cells relative to uninfected and Ad-GFP-infected cells. However, PTEN overexpression significantly attenuated the rate of collagen synthesis regardless of Ang II treatment. Nevertheless, the effect of PTEN was more pronounced in the presence of Ang II (Figure 4(e)).

### 3.4. PTEN Overexpression Regulates Ang II-Induced Matrix Metalloproteinase-2 (MMP-2) and Tissue Inhibitor of Metalloproteinase-1 (TIMP-1) Expression and MMP-2 Activity

**Figure 5:**
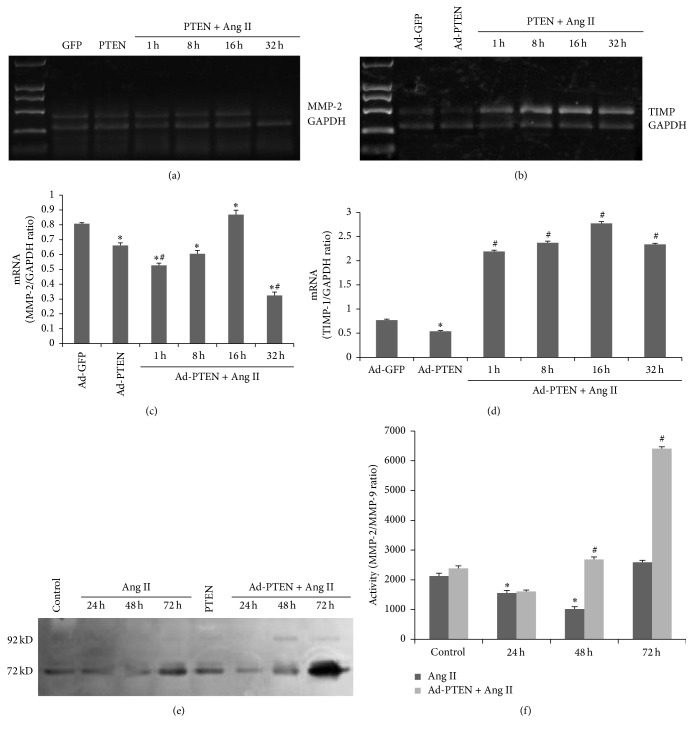
Overexpression of PTEN inhibits Ang II-induced MMP-2 and TIMP-1 production in CFs. After infection for 48 h, fibroblasts were treated with Ang II (10^−7^ mM) for 32 h. (a, b) The MMP-2 mRNA level was assessed by RT-PCR. (c, d) The TIMP-1 mRNA level was assessed by RT-PCR. (e, f) The activity of gelatinase was assessed by gelatin zymography. Each experiment was repeated three times with 3-4 replicates. ^*∗*^ *P* < 0.05, compared with the control Ad-GFP-infected and uninfected cells. ^#^ *P* < 0.05, compared with Ad-GFP + Ang II.

In the presence of Ang II, Ad-PTEN-infected cells showed decreased MMP-2 mRNA at 32 h (Figures 5(a) and 5(c)) and increased TIMP-1 mRNA (Figures 5(b) and 5(d)) at 1, 6, 16, and 32 h after treatment. While MMP-9 protein level was increased in the presence of Ang II at 48 and 72 h in Ad-PTEN-infected cells compared to uninfected cells, MMP-2 protein level was substantially increased at 72 h in Ad-PTEN-infected cells in the presence of Ang II (Figure 5(e)). Gelatin zymography showed that PTEN overexpression increased the ratio of MMP-2 to MMP-9, the protease activity index, in the presence of Ang II (Figure 5(f)).

### 3.5. Effect of PTEN Overexpression on the Phosphoinositide 3-Kinase (PI3K)/Akt Signaling Pathway

**Figure 6:**
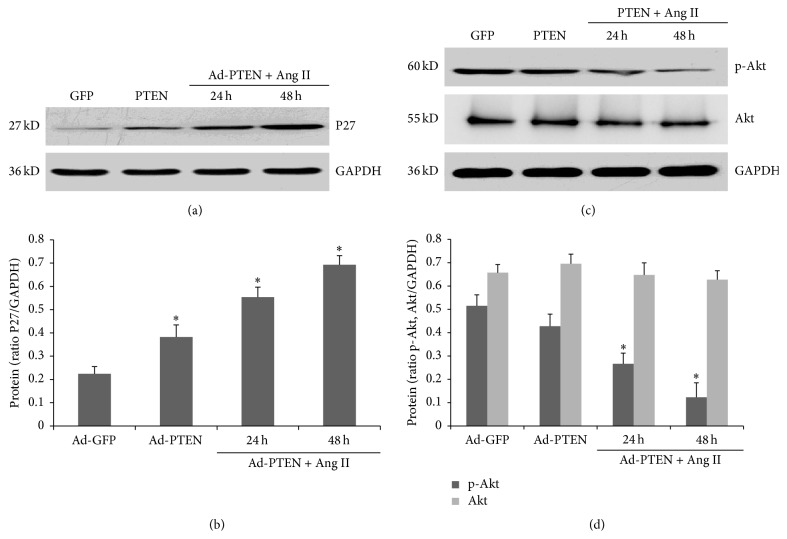
Effect of PTEN on Ang II-induced (10^−7^ mM) Akt and P27 expression in CFs. After infection for 48 h, fibroblasts were treated with Ang II (10^−7^ mM) for 32 h. (a, b) P27 protein expression was assessed by western blotting. (c, d) Total Akt and phospho-Akt (pAkt-308) expression levels were assessed by western blotting. Each experiment was repeated three times with 3-4 replicates. ^*∗*^ *P* < 0.05, compared with the control Ad-GFP-infected and uninfected groups.

To determine the effects of PTEN on Akt/P27 pathway, Akt and P27 expression were analyzed. Compared with Ad-GFP-infected cells, P27 expression was increased in Ad-PTEN-infected cells independent of Ang II stimulation (Figures 6(a) and 6(b)). Moreover, phospho-Akt (pAkt) levels were decreased in PTEN-overexpressing cells independent of Ang II stimulation (Figures 6(c) and 6(d)).

## 4. Discussion

PTEN is widely expressed in the cardiovascular system and plays an important role in cardiovascular disease [7–9]. In the present study, we found that PTEN overexpression not only decreased mRNA levels and protein synthesis rates of collagen types I and III, but also antagonized Ang II-induced collagen synthesis and MMP-2 and TIMP-1 production and gelatinase activity. Moreover, PTEN overexpression decreased pAkt expression and increased P27 expression independent of Ang II stimulation. These results indicate that PTEN could partially elicit its effects in conditions mimicking cardiac fibrosis by regulating collagen metabolism, altering the expression of MMPs, and modulating the Akt/P27 pathway. Detailed mechanistic studies are required to determine whether the effects on collagen metabolism and MMP expression are mediated by the Akt/P27 signaling pathway. Knockdown of endogenous PTEN and stimulation of CFs with Ang II will help identify whether the effect of Ang II on fibrosis is PTEN mediated. Moreover, knockdown of endogenous Akt will also help determine whether Ang II mediates its effect on P27 via Akt.

Mammalian PTEN (molecular weight = 40–50 kDa) is a phosphoinositide 3-phosphatase that directly counteracts growth factor-stimulated PI 3-kinases by metabolizing phosphatidylinositol 3,4,5-trisphosphate (Ptd Ins(3,4,5)P(3)) [18]. PTEN is able to reduce the cellular levels of Ptd Ins (3,4,5)P(3) and antagonize PI 3-kinase signaling. Early studies have shown that PTEN can promote cell cycle arrest and apoptosis as well as inhibit cell motility [18–20]. In the present study, we found that PTEN overexpression in CFs also increased the proportion of cells in the G1/S phase, thereby confirming cell-cycle arrest in the G1/S stage.

The elevation of Ang II concentration is an important cause of cardiac hypertrophy induced by pressure overload. We use Ang II in our experiments to mimic cardiac hypertrophy induced by pressure overload. Ang II stimulation directly decreased the synthesis of PTEN mRNA and protein in cells infected with PTEN-overexpressing adenovirus. The effect of Ang II stimulation on endogenous PTEN mRNA and protein needs to be investigated to confirm the direct effect of Ang II on PTEN expression. Dong et al. demonstrated that downregulation of PTEN expression and activity by Ang II increased proliferation and migration of VSMCs [21]. Thus, Ang II may promote proliferation of CFs if PTEN is downregulated. This hypothesis will need to be confirmed by knocking down endogenous PTEN in CFs and stimulating them with Ang II to test the effects on cell proliferation.

Recent data have identified additional roles of PTEN. Parajuli et al. showed that PTEN regulates cardiac remodeling after myocardial infarction via modulating the Akt/interleukin-10 signaling pathway [10]. Oudit and Penninger have shown that loss of PTEN attenuated the development of pathological hypertrophy and heart failure in response to biomechanical stress [7]. These studies suggest that PTEN participates in functional regulation of the injured heart. Our results show that PTEN overexpression decreases Ang II-induced collagen synthesis, decreases MMP-2 expression, and increases TIMP-1 expression. Thus, PTEN could partially elicit its effects on cardiac fibrosis via its effects in CFs. Parapuram et al. showed that loss of PTEN in dermal fibroblasts causes skin fibrosis [22]. In addition, the dynamic expression of PTEN in rat liver tissues has been found to be negatively correlated with liver fibrosis and activated hepatic stellate cells and to be positively correlated with the reversal of fibrosis [23]. Epithelial PTEN is a crucial gatekeeper that controls acute lung injury and lung fibrosis by modulating alveolar epithelial cell integrity [24]. Thus, we propose the hypothesis that PTEN may play a role in cardiac fibrosis, which might be regulated by collagen metabolism in CFs.

Ang II is known to downregulate P27 expression in the heart. Previous research showed that P27 protein levels were highest in the stationary phase of the cell cycle and began to decline after mitogen-stimulation in cultured myocytes. On completion of the cell cycle, P27 protein accumulated, and cells entered the stationary state [25]. In the current study, P27 expression was increased in Ad-PTEN-infected cells, which led to cell cycle arrest in cardiac fibroblasts. Takashima et al. showed that overexpression of wild-type PTEN in hepatic stellate cells downregulated the Akt, p70 (S6K), and Erk signaling pathways [26]. Furthermore, Li et al. have found that adenovirus-mediated PTEN (AdVPTEN) gene therapy and diamminedichloroplatinum treatment exerted an overlapping effect on the upregulation of P53, P21, P27, Bax, and cleaved caspase-3 in an animal model of small-cell lung cancer [15].

PTEN status is inversely correlated with activation of the oncogenic PI3K/protein kinase B (AKT) pathway in diffuse large B-cell lymphoma cell lines and patient samples, whereas overexpression of PTEN induced cytotoxicity in PTEN-deficient cell lines by inhibiting PI3K/AKT signaling [27]. In skin fibrosis, PTEN-deleted fibroblasts showed elevated Akt phosphorylation and increased expression of connective tissue growth factor (CTGF/CCN2), and PTEN had an inhibitory effect on the PI3K/Akt signaling pathway [22]. These data are in accordance with the current study, which demonstrated phospho-Akt levels were decreased in PTEN-overexpressing cells. Ang II stimulation decreased the expression of PTEN, and the inhibitory effect of PTEN on PI3K/Akt was eliminated, which increased the synthesis of collagen. Furthermore, PTEN overexpression decreased Ang II-induced collagen synthesis. Thus, the effects of PTEN on cardiac fibrosis could at least partially be mediated via regulation of the PI3K/AKT/P27 pathway. However, additional molecular studies by PTEN knockdown in neonatal CFs will be required to elucidate the function of PTEN in conditions of cardiac fibrosis.

A potential limitation of the present study was that Akt and p27 were not inhibited, precluding analysis of the direct role of PTEN on the Akt/P27 pathway. Because of the in vitro nature of the study, we could not clarify the role of PTEN in cardiac fibrosis. These mechanisms should be investigated in future studies.

## 5. Conclusions

In conclusion, PTEN could regulate collagen metabolism of neonatal rat CFs via activation of the Akt/P27 pathway. Elucidation of the mechanism of action will help thus providing a new possible target for the treatment of cardiac fibrosis.

## Figures and Tables

**Table 1:** Primers designed and used for the RT-PCR studies.

Gene	Primers	Product size (bp)	Annealing temperature
PTEN	Forward 5′-AGAACTTATCAAACCCTT-3′	186	55°C
	Reverse 5′-GTCCTTACTTCCCCAT-3′		

Col I-*α*1	Forward 5′-CTCAGGGGCGAAGGCAACAGT-3′	125	50°C
	Reverse 5′-ATGGGCAGGCGGGAGGTCT-3′		

Col III-*α*1	Forward 5′-ATGGTGGCTTTCAGTTCAGC-3′	425	45°C
	Reverse 5′-TGGGGTTTCAGAGAGTTTGG-3′		

MMP-2	Forward 5′-TGG TCGCAGTGATGGCTTCCTCT-3′	414	57°C
	Reverse 5′-CCCCACTTCCGGTCATCATCGTAG-3′		

TIMP-1	Forward 5′-TCGACGCTGTGGGGAATG-3′	466	54°C
	Reverse 5′-AAAGAACGGAGGAAACAG-3′		

GAPDH	Forward 5′-TGCTGAGTATGTCGTGGAGT-3′	289	55°C
	Reverse 5′-AGTCTTCTGAGTGGCAGTGAT-3′		

**Table 2:** Effect of PTEN overexpression on the cell cycle in CFs.

	G1	S	G1/S
Ad-PTEN	76.4%	8.07%	9.47^*∗*^
Ad-GFP	59.4%	24.5%	2.42
Untreated group	61.8%	21.6%	2.86

^*∗*^ *P* < 0.05, compared with the control Ad-GFP and untreated groups.
